# Multi-species prey dynamics influence local survival in resident and wintering generalist predators

**DOI:** 10.1007/s00442-021-05042-2

**Published:** 2021-09-22

**Authors:** Daniel Oro, Ana Sanz-Aguilar, Francesc Carbonell, Joan Grajera, Ignasi Torre

**Affiliations:** 1 Theoretical and Computational Ecology Group, Center for Advanced Studies of Blanes (CEAB-CSIC), Accés Cala Sant Francesc 14, 17300 Blanes, Spain; 2 Animal Demography and Ecology Unit, IMEDEA (CSIC-UIB), Miquel Marques 21, 07190 Esporles, Spain; 3 Applied Zoology and Conservation Group, University of the Balearic Islands, Crtra. Valldemossa s/n, 07122 Palma, Spain; 4 Catalan Ornithological Institute, Girona 168, 08037 Barcelona, Spain; 5 BiBio Research Group, Natural Sciences Museum of Granollers, Francesc Macià 51, 08402 Granollers, Spain

**Keywords:** Survival, Small mammals, Wintering ecology, Generalist raptor, Top-down control

## Abstract

Stochasticity in food availability influences vital rates such as survival and fertility. Life-history theory predicts that in long-lived organisms, survival should be buffered against environmental stochasticity showing little temporal variability. Furthermore, to optimize survival prospects, many animal species perform migrations to wintering areas where food availability is larger. Species with large latitudinal distribution ranges may show populations that migrate and others that are resident, and they may co-occur in winter. One example of these species is the predatory raptor buzzard *Buteo buteo*. Here, we test whether temporal variability in the density of five small mammal species of prey inhabiting different habitats (shrubland and forests) influences local annual survival of buzzards in a wintering area depending on their age and residency status (residents versus wintering individuals). We found that prey density explained a considerable amount of annual changes in local survival, which was higher for older and resident birds. This difference in local survival likely corresponded to philopatry to the wintering area, which was larger for residents and increased when prey density was larger. The total density of prey inhabiting open shrublands was the variable explaining more variance in temporal variability of local survival, even though the study area is mostly occupied by woodlands. Temporal population dynamics of the different small mammals inhabiting shrublands were not synchronous, which suggests that buzzards preyed opportunistically on the most abundant prey each winter. Generalist predation may buffer the impact of resource unpredictability for pulsed and asynchronous prey dynamics, typical of small mammals in winter.

**Supplementary Information:**

The online version contains supplementary material available at 10.1007/s00442-021-05042-2.

## Introduction

The importance of food availability for most vital rates, such as recruitment, survival and fertility, depends on life-history strategies. For short-lived species, stochastic variability in food availability and predators largely influence their population dynamics (bottom-up and top-down regulation, respectively) (Hanski et al. 1993; Frederiksen et al. 2006). For long-lived animals lacking predators, fluctuations in prey availability, in the absence of additive mortality, regulate their population dynamics by density-dependence (Saether and Bakke 2000; Millon et al. 2019). Life-history theory predicts that survival of long-lived organisms tends to be buffered against environmental stochasticity, whereas other traits such as recruitment, skip breeding and mainly fertility are more sensitive to this stochasticity (Lande et al. 2003; Karell et al. 2009). Raptors are among these species, being both top predators and long-lived organisms. In recent years, numerous raptor studies have estimated their survival rates and how they vary as a function of individual covariates (e.g., sex, age) for several species (see review in Newton et al. 2016). Survival, especially those of adult birds, tends to be constant over the years, and this pattern is altered mainly by anthropogenic impacts on survival (Sergio et al. 2011; Martínez-Abraín et al. 2012; Tavecchia et al. 2012; Badia‐Boher et al. 2019). Nevertheless, the ecological processes affecting raptor survival, and more particularly the density of prey, remain little known. This is particularly true because most raptors are territorial and sample sizes are often small, which represents a challenge to assess the ecological processes influencing temporal fluctuations in survival. Proxies of food availability, such as global climatic and vegetation cover indexes, have been also occasionally used to test their influence on survival (Grande et al. 2009; Mihoub et al. 2010). Furthermore, stochasticity in prey densities may occur over time (seasonality in prey fluctuations) and space (spatial heterogeneity in prey densities) and this may influence spatio-temporal variability in raptor survival (McClure et al. 2020). A paradigmatic example is cycles in small mammal densities and their influence on survival of predators in high-latitude ecosystems (Brommer et al. 2002; Karell et al. 2009; Millon et al. 2014). Spatio-temporal variability in survival may be particularly acute also for long-lived migrant birds moving in large geographical areas, and selecting a suitable wintering area may optimise survival probabilities and fitness prospects (Harris et al. 2005; Genovart et al. 2013; Klaassen et al. 2014; Sergio et al. 2014b). Yet, some species may have distinct populations with strategies being either resident or migrant and with consequences for population heterogeneity in individual survival and population dynamics (Sanz-Aguilar et al. 2012, 2015). Examples of these species are those with mainland and island populations, with large distribution ranges, and with opportunistic habits for exploiting anthropogenic food subsidies (such as wolves, kites, gulls, and storks). Here, we test whether temporal variability in the density of five small mammal potential prey species influences local annual survival of common buzzards *Buteo buteo* in a Mediterranean wintering area depending on their age and residency status (i.e., residents versus wintering birds). These small mammals have varying seasonal life cycles and inhabit different habitats (shrubland and forests) (Diaz et al. 2010; Torre et al. 2020). Since buzzards are opportunistic predators (Graham et al. 1995; Reif et al. 2004), we expect that local survival will be influenced by the density of the commonest prey species each year. We also expected higher dependency on small mammals in autumn–winter, owing that alternative prey such as reptiles and insects commonly consumed by buzzards during the breeding season in this area are unavailable. Small mammal densities in winter are higher in open habitats (Torre et al. 2018), thus we will also test the hypothesis that small mammals inhabiting forests should have a lower influence on local survival than those occupying open habitats, where buzzards in the study area are commonly hunting (Grajera and Carbonell 2017).

## Methods

### Study area

**Fig. 1:**
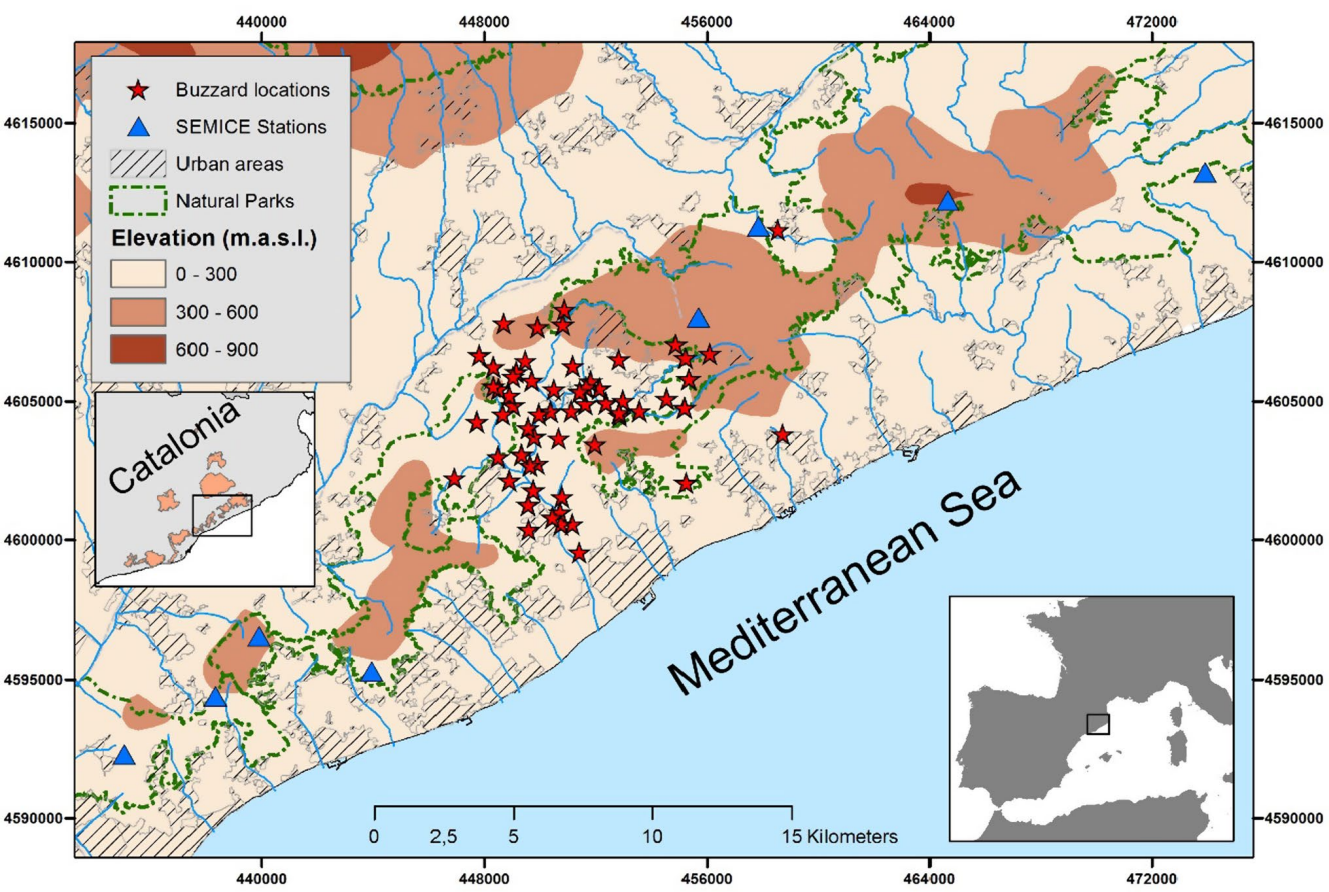
Map of the study area in Barcelona province (north-eastern Spain) showing the locations where buzzards were trapped and marked and the locations (eight stations) where small mammals were sampled and their average densities calculated

The study area is ca. 50 km^2^ and is located over a coastal mountain range (Barcelona, Catalonia, NE Spain, Fig. 1). Habitat is mainly composed of dense forests of pines (mainly *Pinus pinea*) and oak trees (mainly *Quercus ilex*), with patches of riverside forests associated with small streams, cereal, and set aside fields, orchard, and some urban areas (altitude: 79–380 m.a.s.l.). Forests and open habitats (shrublands and crops) occupy ca. 73 and 18% of the surface, respectively, with the remaining 9% covered by urban areas. In the study area, buzzards triple in density during winter due to the arrival of wintering individuals, compared to the density recorded during the breeding season (Grajera and Carbonell 2017).

### Capture field protocols for buzzards and small mammals

From 2009 to 2019, we marked 111 wintering visitors (66 first winter and 45 older birds) and 36 residents (7 first winter–marked as chicks, see below, and 29 adults breeding in the area) buzzards. Birds were caught depending on the circumstances (mist nets, noose traps, Swedish hawk traps, and crossbow netting). Chicks born in the area were marked at the nests, which were previously located during the spring fieldwork season. We found sixteen breeding territories within the study area (and five additional territories that partially overlap their boundaries within this area), which shows that the density of breeding buzzards was high (ca. one territory each 3.1 km^2^). We assumed that we did not miss any breeding territory in this study area, owing that breeding buzzards are territorial and perform territorial flights over the nests, which are very conspicuous. Residents were marked mostly in their territories during breeding, and wintering birds were marked from late autumn to late winter (October–February). Resident birds were considered “marked” in the capture–recapture matrix when first resighted or recaptured during the wintering season to ensure that first-year local survival of all marked birds corresponded to the same amount of time. Birds were marked using a colour wing-tag in each wing, a metal ring in one leg, and a colour ring in the other leg with an individual alphanumeric code. To assess tag loss, we used different methods combined: the three types of marks (four marks in total), the plumage features (buzzards show high individual plumage variability), the moult pattern, and the high number of detailed observations of each individual mostly while perching on poles (> 90% from total observations) (mean = 33 observations, median = 11, range = 2–441). During the study, tag loss was anecdotal (only one individual confirmed) and not considered in our capture-recapture modelling. Resident birds were distinguished from wintering visitors partially by their plumage and the intensive monitoring of the breeding population in the study area (Grajera and Carbonell 2017). This monitoring included 27 breeding events monitored over the study by remote cameras with motion sensor for the study of the diet in a number of nests (average: 3.4 nests/year, range: 1–4) (unpublished data). Cameras were set for long periods (11 days ± 13 SD per nest, *n* = 279 camera-days) and allowed us to confirm the residency status of adults caught out of the breeding season. Resights of wing-tags from the distance allowed us the identification of breeding resident birds not monitored using nest-cameras in the study area. Furthermore, for chicks marked in the nest and adults trapped within their breeding territories, their origin was certain. Plumage was categorized in three categories: dark (84% of caught birds), intermediate (10%) and white (6%). We assumed that plumages of the two later categories corresponded to wintering birds coming from northern latitudes; all breeding birds marked and chicks marked in the nest and caught in the first winter during the study showed dark plumages. Nevertheless, we cannot rule out that some non-resident birds with dark plumages originated from northern latitudes or relatively close areas. In any case, we categorized the individuals as residents and wintering visitors, this last category including birds from both northern latitudes and closer areas. Age was assigned using EURING methodology and plumage characteristics following previous studies from southern Europe (Zuberogoitia et al. 2005) and due to the small sample size, only two groups were defined: birds in their first winter (including birds born in the study area and marked at the nest) and older birds. During each winter, resightings of marked birds were performed by car and walking transects over the study area, as well as using bait camera trapping and trapping for specific cases. We spent an average of 8 h each week Trapping allowed us to recapture some marked birds that were difficult to resight due to their tendency to hunt in woodlands. The effort of marking and resighting was kept relatively constant for the sampling winter periods over the years (mean = 4.8 days/week; SD = 1.7).

Small mammal relative densities and their annual variability were recorded using a standard grid-trapping long-term monitoring scheme (Torre et al. 2018). Sampling was performed each autumn (October–December) from 2009 to 2019, following the SEMICE monitoring scheme (Torre et al. 2018). Sampling plots were set in grids to include 36 traps (6 × 6 trapping scheme) spaced 15 m, alternating in position 18 Sherman traps (Sherman Co., USA) with 18 Longworth traps (Penlon Ltd., Oxford, UK). Traps were baited with food and insulated by including hydrophobic cotton for bedding. Traps were operated during three consecutive nights and revised each day during the early morning. Standardized sampling effort allowed establishing the relative abundance of common small mammal species in buzzard’s territories, by gathering the information recorded in eight nearby stations to the study area (Fig. 1). Stations were placed in two types of contrasted habitats: forests and shrublands-grasslands (Mediterranean maquis of *Quercus coccifera* and other fire-adapted resprouting species). These two contrasting habitats showed strong differences in suitability for small mammals regarding several abiotic and biotic factors (Torre et al. 2020). Population relative densities were obtained using TRIM software for the analysis of time series of counts with missing observations (Pannekoek and Van Strien 2005). Distance from the centre of the buzzard study area to small mammal sampling stations ranged between 5 and 24 km. Densities of small mammals showed spatial synchrony within years and between habitats (Diaz et al. 2010; Stefanescu et al. 2020; Torre et al. 2020). Thus, overall annual differences in densities reflected actual temporal changes in small mammal availability to buzzards. Species sampled in the study area were white-toothed shrew (*Crocidura russula*), Algerian mouse (*Mus spretus*), wood mouse (*Apodemus sylvaticus*)*,* Yellow-necked mouse (*Apodemus flavicollis*), and bank vole (*Myodes glareolus*). The species with higher densities were shrew, Algerian mouse, and wood mouse (see “Results”). Despite other small mammal species being present in the area, these three species were considered as keystone prey, representing the bulk of the diet for other generalist forest-open predators in the study area (70–75% of occurrence among all small mammals in common genet and barn owl diets) (Torre et al. 2018). We performed GLMM models with negative binomial error (to avoid overdispersion (Zeileis et al. 2008) to test whether the relative density of the three most abundant species of small mammals was explained by habitat categorization (forests and shrublands–grasslands), and a visual inspection of data was used in the case of the two rarest species. Sampling station was added to the models as a random factor.

To assess whether population relative densities of the five species of small mammals were synchronous over the years (i.e., their fluctuations were correlated), we compute community-wide synchrony and its significance via Monte Carlo randomizations using the R-package ‘synchrony’. Values of community-wide synchrony range between 0 and 1, and they would have the maximum value when all species fluctuate in parallel. The Monte Carlo randomizations are performed by shuffling the columns of the community matrix independently, and randomizations also return the mean correlation between the columns of the matrix.

### Capture–recapture modelling of local survival

We modelled the annual probabilities of buzzard survival and fidelity to the wintering area (i.e., local survival) using capture–recapture models for open populations (Lebreton et al. 1992). We created annual encounter histories for every marked bird in which individuals captured/resighted during the winter period (October year *t* to February year *t* + *1*) were pooled in a single occasion, resulting in 347 encounters of 147 marked birds. We first tested the goodness-of-fit of the general Cormack–Jolly–Seber model (CJS hereafter) by age at first capture (first winter vs older birds) and residency status groups (i.e. residents and wintering birds) using the program U-CARE (Choquet et al. 2009). Then, using program MARK (White and Burnham 1999) and starting with a general model with survival probabilities varying over time between residency status groups (but in parallel between age classes within a group), we first tested the effect of time and residency status on detection probability. Once the best structure for this parameter was selected, we modelled local survival. We evaluated if local survival was related to residency status, age, time and/or prey abundance using the mean density of small mammals in the area in late autumn as a temporal covariate. In particular, we tested nine covariates accounting for annual variability of small mammal density in the study area: (a) for each of the five species; (b) for shrubland species (*C. russula* and *M. spretus*); (c) for shrubland species plus *A. sylvaticus,* which occupies both habitats; (d) for species occupying forests (*A. flavicollis* and *M. glareolus*); (e) finally for all the species together.

Model selection was based on Akaike Information Criterion corrected by sample size (Burnham and Anderson 2002). Models differing by < 2 AICc points were considered equivalent and the Akaike weights were calculated as an index of model plausibility over the models performed. The statistical significance of temporal covariates was assessed using analysis of deviance with a Fisher–Snedecor distribution (ANODEV; (Grosbois et al. 2008). The percentage of temporal variation in local survival explained by densities of small mammals *R*^2^ was calculated by comparing deviance of models with covariate (*Dev*_*dens*_) to the constant (*Dev*_*const*_) and the time-dependent models (*Dev*_*t*_), such that:

$$ R^{2} = \frac{{{\text{Dev}}_{{{\text{const}}}} - {\text{Dev}}_{{{\text{dens}}}} }}{{{\text{Dev}}_{{{\text{const}}}} - {\text{Dev}}_{{\text{t}}} }}. $$

## Results

**Fig. 2:**
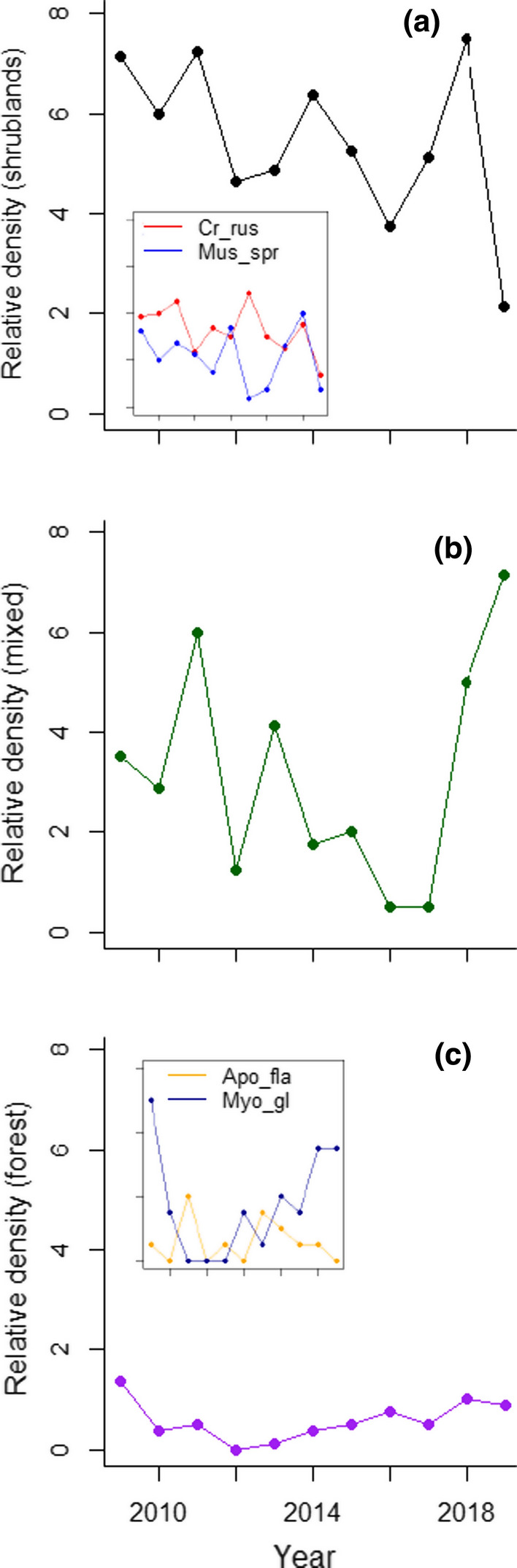
Annual variability (2009–2019) of the relative density of the five species of small mammals for each habitat categorization: **a** only shrublands (inset show densities separately for *Crocidura* and *Mus*); **b** shrubland and forest habitats, corresponding to *A. sylvaticus*; and **c** only forests (inset shows densities separately for *A. flavicollis* and *M. glareolus*). All panels have the same scales except inset for forest species (maximum relative density = 1.5). Panels show annual values averaged for the eight trapping stations

**Fig. 3:**
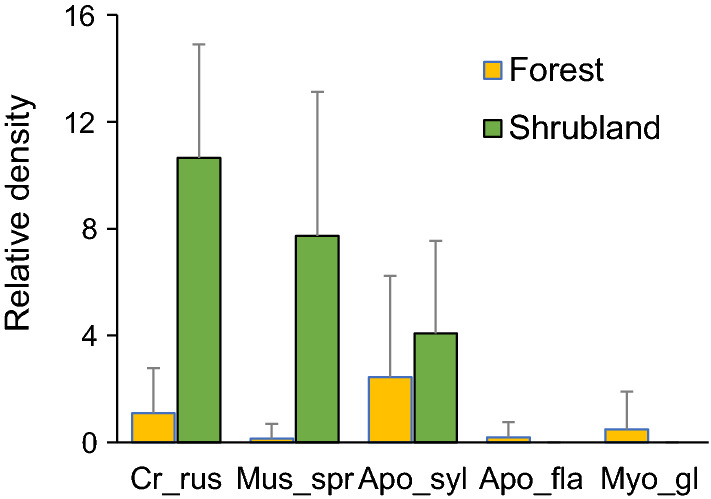
Mean relative density (with ± S.D.) of each small mammal species at forest and shrubland sampling stations in the study area during 2009–2019 (see Fig. 1). *Apo_fla* Yellow-necked mouse, *Apo_syl* wood mouse, *Cr_rus* white-toothed shrew, *Mus_spr* Algerian mouse,* Myo_gl* bank vole

During the study period (autumn 2009-autumn 2019) we trapped 827 small mammal individuals of five species. The white-toothed shrew was the more abundant (36.2%), followed by wood mouse (33.4%) and Algerian mouse (22.7%). Bank vole and Yellow-necked mouse represented < 6% of captures. Mean weight for the trapped individuals over the study was 8.1 g (SD = 1.2) for shrews, 13.8 g (SD = 3.5) for Algerian mice, 24.2 g (SD = 6.5) for wood mice, 26.4 g (SD = 3.5) for yellow-necked mice and 23.9 g (SD = 3.8) for bank voles. Relative densities of small mammals estimated during late autumn over 104 sampled trapping occasions from 2009 to 2019 varied differently for each species over the years (Fig. 2). On average, total densities were higher in shrublands than in forests (mean = 21.6, S.D. = 6.6; mean = 5.3, S.D. = 5.3, respectively) (Fig. 3). Species were assigned to preferred habitats considering the specific responses to land-use change in the study area (Torre et al. 2015): white-toothed shrew and Algerian mouse mostly inhabited open habitats, whereas wood mouse, Yellow-necked mouse, and bank voles were forest species. GLMM models showed that relative densities of white-toothed shrews and Algerian mice were positively influenced by shrublands (*R*^2^ = 0.51 and *R*^2^ = 0.28, respectively), whereas the density of wood mice was not higher in this habitat (*R*^2^ = 0.03). Bank voles and yellow-necked mice were only trapped in forests. AIC values showed that GLMMs with negative binomial showed better fit than Poisson, and solved the problem of overdispersion in all cases (Supplementary Material, Table S1). Fluctuations of population density over the years for the five species of small mammals were not significantly correlated (community synchrony: 0.3535; mean pairwise correlation: 0.07458; community synchrony p value (one-tailed test [greater]): 0.1667).

### Capture-mark recapture modelling of buzzard survival

During the study, we recorded 5488 observations of marked birds that could be identified. From these, 2572 resightings corresponded to the wintering sampling periods (winter mean number of resights = 234; range = 55–386). Most resightings (84%) corresponded to resident birds, since they were observed more often within each wintering sampling period.

**Fig. 4:**
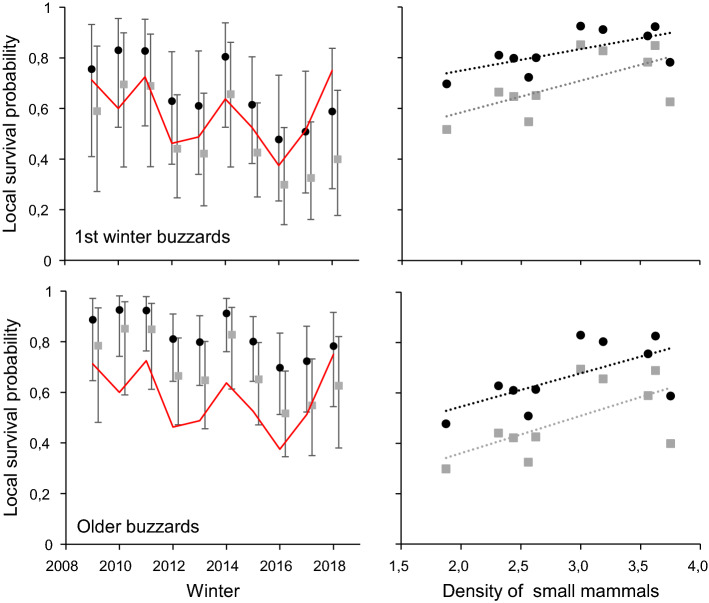
Left panels: annual estimates (and 95% CI) of local survival probabilities of the first winter and older resident (circles) and wintering (squares) buzzards from winter 2009–10 until winter 2019–20 (Model Survival (age + g + time) Resight (.), not shown in Table 1 since ΔAICc = 9.11). The red dashed line indicates the estimated relative density of shrubland small mammals (*M. spretus* and *C. russula*) during the previous autumn. Right panels: relationship between annual estimates of local survival probabilities of the first winter and older resident (circles) and wintering (squares) buzzards and the annual density of small mammals

The overall test of goodness-of-fit of the CJS model was not statistically significant indicating that the CJS model by residency and age groups can be used as a starting model (${\chi }_{50}^{2}$= 29.90, *p* = 0.989). Lack of a statistically significant transient effect suggested that the frequency of birds just migrating through the study area was low and that our assignment of residency status was not biased. Model selection indicated that models with constant resighting probabilities or resighting probabilities depending on the wintering status of birds were equally supported and preferred over models including temporal variation (Table 1). In fact, both groups of birds showed similar resighting probabilities, being slightly higher for residents (0.83, 95% CI = 0.72–0.90) than for wintering visitors (0.79, 95% CI = 0.69–0.87) (Model 4, Table 1). A model considering a constant survival for older residents (model 13) was not retained. Both constant and group dependent resight formulations were used to model local survival, and, in this case, models including additive differences between first winter and older residents and wintering visitors were preferred (Table 1). Mean local survival of resident buzzards (1st winter residents = 0. 68, 95% CI = 0.50–0.81; older residents = 0.81, 95% CI = 0.72–0.87) was higher than mean local survival of wintering birds (1st winter visitors = 0.50, 95% CI = 0.37–0.63; older visitors = 0.67, 95% CI = 0.59–0.74) (Model 11, Table 1). Models with constant survival during the study period were better ranked than models with full temporal variations. However, up to 52% of temporal variation in local survival probabilities was explained by the total density of shrubland small mammal species (*M. spretus* and *C. russula*) during autumn (Table 1, Fig. 4). Best models included the effect of the small mammal density covariates, which were statistically significant when we tested the density of the species of open shrubland habitats, but also for the species of mixture habitats and the total density of all species. The covariate was not significant when the density of single small mammal species or only the density of forest species was considered (see Table 1). Buzzards have been described as predators in open habitats. However, our study area is mostly covered by forests, and observing buzzards hunting in this habitat is challenging. Our results confirm that buzzards preferred hunting in shrublands and avoided forests. Local survival and small mammal density showed a positive relationship (see Fig. 4 for the best model).

**Table 1:** Model selection testing the effects of year (t), residency status groups (g), age (first winter vs. older birds) and small mammal relative density (D) on local survival probabilities of Common buzzards

Model	Survival	Resight	Np	Dev	ΔAICc	*Wi*	*R*^2^	*F*_(1,8)_	*p* value	β slope covariate (SE)	
1	Age + g + D_(*Mus_spr* + *Cr_rus*)_		5	279.25	0	0.25	0.52	8.67	0.019	0.79 (0.29)	
2	Age + g + D_(Mus_spr + Cr_rus + Apo_syl)_		5	279.75	0.50	0.19	0.49	7.68	0.024	0.49 (0.19)	
3	Age + g + D_(all species)_		5	280.56	1.31	0.13	0.48	6.30	0.036	0.76 (0.31)	
4	Age + g + D_(*Mus_spr* + *Cr_rus*)_	g	6	278.88	1.71	0.11	0.52	8.78	0.018	0.79 (0.30)	
5	Age + g + D_(*Mus_spr* + *Cr_rus* + *Apo_syl*)_	g	6	279.35	2.18	0.08	0.49	7.83	0.023	0.49 (0.19)	
6	Age + g + D_(*Apo_syl*)_		5	281.77	2.52	0.07	0.37	4.63	0.064	0.21 (0.09)	
7	Age + g + D_(all species)_	g	6	280.16	2.99	0.06	0.46	6.95	0.029	0.77 (0.32)	
8	Age + g + D_(*Apo_syl*)_	g	6	281.37	4.20	0.03	0.37	4.72	0.062	0.21 (0.10)	
9	Age + g + D_(*Mus_spr*)_		5	284.81	5.56	0.02	0.18	1.78	0.219	0.21 (0.13)	
10	Age + g + D_(*Cr_rus*)_		5	285.70	6.45	0.01	0.13	1.17	0.310	0.24 (0.17)	
11	Age + g		4	287.80	6.49	0.01					
12	Age + g + D_(*Mus_spr*)_	g	6	284.47	7.30	0.01	0.18	1.77	0.220	0.21 (0.13)	
13	Age + g + D_(*Mus_spr* + *Cr_rus*)_/Ad_res_		5	286.72	7.47	0.01	0.04	0.32^a^	0.585	0.25 (0.25)	
14	Age + g + D_(*Cr_rus*)_	g	6	285.31	8.14	0.00	0.13	1.19	0.307	0.24 (0.17)	
15	Age + g	g	5	287.43	8.18	0.00					

*np* number of estimable parameters, *Dev* relative deviance, *AICc* Akaike’s information criterion adjusted for small sample size (c), *ΔAICc* difference between current model and the model with the lowest AICc, *Wi* Akaike weight of model *I*, ‘.’ *I* no effect, i.e., constant parameter; ‘ + ’ additive effect; ‘*’ *I*  interaction. *Apo_fla* Yellow-necked mouse, *Apo_syl* wood mouse; *Cr_rus* white-toothed shrew; *Mus_spr* Algerian mouse; *Myo_gl* bank vole. *Ad*_*res*_ resident adults with constant local survival. Only the first 15 best-ranked models are shown (Supplementary information, Table S2)

^a^Here, degrees of freedom are *F*_(1,9)_

## Discussion

Testing how stochastic fluctuations of prey densities influence survival of long-lived predators is challenging mainly due to the difficulties for collecting robust data simultaneously for prey and predators at suitable spatio-temporal scales (Oro and Furness 2002; Karell et al. 2009; Margalida et al. 2014). Yet, for non-colonial long-lived birds, sample sizes tend to be small and the power to assess the ecological processes influencing their survival is limited. From a methodological point of view, we cannot be completely certain about the residency status of all marked birds in the sample, although the intense monitoring of breeding territories may have greatly reduced this potential bias. Despite these constraints, we found that the annual variability in the density of small mammals (mice, shrews, and voles), which are the main prey of buzzards in this region during winter (Mañosa and Cordero 1992), influenced their local survival at the wintering area. As expected for long-lived raptors, juvenile birds showed lower local survival than older birds (Kenward et al. 1999; Morrison 2003; Sanz-Aguilar et al. 2015). While the estimate of local survival for resident birds may be close to the actual survival value (especially for adults that seldom emigrate to other breeding areas), the local survival for wintering birds likely reflects their tendency to return to the wintering area, which is lower than for residents. We cannot disentangle whether this lower survival is only due to a higher permanent dispersal of wintering birds (which in models is confounded with mortality) or there is also actual higher mortality, both processes influenced by competition with residents and likely with other similar predators. At the study wintering area, residents (i.e., breeding adults and local-born juveniles) and wintering visitors coming from abroad co-occur and buzzards triple in density (Grajera and Carbonell 2017). Whatever the reason for this lower survival, we show that wintering buzzards would be more philopatric to the wintering area when they found high prey densities that year, which likely reduces the strength of density-dependence (Oro et al. 2006; Ross et al. 2015; Radchuk et al. 2016). Furthermore, compared to the surrounding areas, annual rainfall here is higher (> 800 mm) and rainfall is associated with larger densities of small mammals (Diaz et al. 2010, p. 201). This likely increases habitat suitability of the study area for wintering buzzards over the years. For migratory animals, wintering areas are crucial for their survival and population dynamics, since winter is a particularly challenging season with harsher climatic conditions and higher competition for food (Grande et al. 2009; Hinnebusch et al. 2010; Wellicome et al. 2014; Sergio et al. 2014b; Baltag et al. 2018; Millon et al. 2019). Furthermore, studying winter survival of species having resident populations and migrant populations, such as buzzards, have great interest to assess the ecological and evolutionary effects of a warming climate and the shift in distribution ranges on wintering strategies (Paprocki et al. 2014; Martín et al. 2014; Sanz-Aguilar et al. 2015). It remains unknown the consequences of the different local survival for resident and wintering birds for their population dynamics, but it is expected that these dynamics are influenced by environmental stochasticity and the changes caused in prey densities over the winter seasons (Sherry and Holmes 1995). Wintering birds are likely searching for areas where prey densities and chances for surviving are higher, but in these areas, intra- and interspecific competition maybe also larger (Johnson 2007). Small mammals are the main prey for a diverse community of predators in the study area, including other raptor species (both diurnal and nocturnal) and mesocarnivores (Torre et al. 2013). Furthermore, predation rates on small mammals change along environmental gradients, show spatial association but a small spatial overlap between species of predators, which suggests they avoid interspecific competition (Torre et al. 2013).

The population dynamics of the small mammal community shed light on the ecological and evolutionary demography of buzzards in wintering areas. First, not only the survival of juveniles is affected by prey density every winter, but also that of older birds, which should be more buffered against environmental stochasticity (Gamelon et al. 2017). As mentioned earlier, winter is the season when density-dependence is stronger due to the presence of individuals born in the last cohort and the occurrence of harsher climatic events (Sanz-Aguilar et al. 2015, 2016). Furthermore, we found that small mammals in the study area have asynchronous temporal dynamics, and none of the species, when considering their single fluctuations, explained part of the observed variability in survival. Total fluctuations of the more abundant species occupying open habitats, where buzzards are mostly observed while hunting in winter, are influencing local survival and the trend to return to the wintering area. Wintering buzzards take profit of the higher availability of shrews in autumn–winter (Torre et al. 2020), when the more widespread and abundant wood mouse is relatively scarce (Stefanescu et al. 2020). Further, buzzards surely benefitted from hunting more diurnal small mammals such as white-toothed shrew and Algerian mouse, which show polyphasic rhythm and diurnal activity, respectively, in winter (Palomo et al. 2009). This also suggests that buzzards preyed opportunistically on the commonest prey each winter and they do not target a single species despite their differences in body mass. Generalist predation may buffer the impact of resource unpredictability for pulsed and asynchronous prey dynamics, typical of small mammals in winter (Hanski et al. 1991; Yang et al. 2008; Fargallo et al. 2009). At the same time, our results suggest the capability of generalist predators to exert top-down forcing on lower trophic levels and community dynamics of their prey (Sergio et al. 2014a).

## Supplementary Information

Below is the link to the electronic supplementary material.

Supplementary file1 (PDF 570 KB)
