# Supplementary material for: Multi-species prey dynamics influence local survival in resident and wintering generalist predators
Source: Oecologia. 2021 Sep 22;197(2):437–46. doi: 10.1007/s00442-021-05042-2 (PMC8505301; doi:10.1007/s00442-021-05042-2)
Supplement: Supplementary file 1 — Supplementary file1 (PDF 570 KB) [file 442_2021_5042_MOESM1_ESM.pdf]

Supplementary Information

Table S1. Best mixed models (using either Poisson or Negative Binomial distributions) for the categorical predictor (habitat) on relative density of the three most abundant species of small mammals. Standardized estimates are given for fixed effects of the models and the SE of the estimates in parenthesis. P(z) indicates significant estimates.

	A. sylvaticus		C. russula		M. spretus		Total	
	Poisson	NBin	Poisson	NBin	Poisson	NBin	Poisson	NBin
(Intercept)	0.93^{***}	0.95^{***}	-0.22	-0.22	-1.96^{***}	-1.96^{***}	1.50^{***}	1.50^{***}
	(0.21)	(0.19)	(0.34)	(0.34)	(0.30)	(0.31)	(0.18)	(0.18)
Open habitat			2.56^{***}	2.56^{***}	4.00^{***}	4.00^{***}	1.55^{***}	1.54^{***}
			(0.62)	(0.62)	(0.31)	(0.35)	(0.36)	(0.36)
Log Likelihood	-302.2	-227.27	-192.46	-189.44	-131.50	-112.54	-336.76	-301.43
AICc	608.57	460.77	391.16	387.28	269.24	233.48	679.76	611.25
R^2 marginal	0.10	0.03	0.60	0.51	0.96	0.28	0.60	0.44
R^2 conditional	0.46	0.07	0.87	0.72	0.96	0.28	0.85	0.59
Residual deviance	367.26	110.07	162.03	124.16	155.84	75.88	291.40	120.68
Ratio	3.63	1.09	1.60	1.23	1.54	0.75	2.88	1.19
Num. obs.	104	104	104	104	104	104	104	104

$^{***}p < 0.001$; $^{**}p < 0.01$; $^{*}p < 0.05$

Table S2. Complete table for model selection testing the effects of year (t), residency status groups (g), age (first winter vs. older birds) and small mammal relative density (D) on local survival probabilities of Common buzzards. Notation: np = number of estimable parameters, Dev = relative deviance; AICc = Akaike's information criterion adjusted for small sample size (c); ΔAICc = difference between current model and the model with the lowest AICc; Wi = Akaike weight; '.' I=no effect, i.e. constant parameter; '+' =additive effect; '*' i= interaction. *Apo_fla* = Yellow-necked mouse; *Apo_syl* = wood mouse; Cr_rus = white-toothed shrew; *Mus_spr* = Algerian mouse; *Myo_gl* = bank vole. Ad_{res} = resident adults with constant local survival.

Model	Survival	Resight	Np	Dev	AICc	ΔAICc	Wi	R^2	$F_{(1,8)}$	p-val
1	age+g+D$_{(Mus_spr + Cr_rus)}$	.	5	279.25	562.22	0	0.25	0.52	8.67	0.019
2	age+g+D$_{(\text{Mus_spr + Cr_rus + Apo_syl})}$	.	5	279.75	562.72	0.50	0.19	0.49	7.68	0.024
3	age+g+D$_{(\text{all species})}$	.	5	280.56	563.53	1.31	0.13	0.48	6.30	0.036
4	age+g+D$_{(Mus_spr + Cr_rus)}$	g	6	278.88	563.93	1.71	0.11	0.52	8.78	0.018
5	age+g+D$_{(Mus_spr + Cr_rus + Apo_syl)}$	g	6	279.35	564.40	2.18	0.08	0.49	7.83	0.023
6	age+g+D$_{(Apo_syl)}$	.	5	281.77	564.74	2.52	0.07	0.37	4.63	0.064
7	age+g+D$_{(\text{all species})}$	g	6	280.16	565.21	2.99	0.06	0.46	6.95	0.029
8	age+g+D$_{(Apo_syl)}$	g	6	281.37	566.42	4.20	0.03	0.37	4.72	0.062
9	age+g+D$_{(Mus_spr)}$	.	5	284.81	567.78	5.56	0.02	0.18	1.78	0.219
10	age+g+D$_{(Cr_rus)}$	.	5	285.70	568.67	6.45	0.01	0.13	1.17	0.310
11	age+g	.	4	287.80	568.71	6.49	0.01			
12	age+g+D$_{(Mus_spr)}$	g	6	284.47	569.52	7.30	0.01	0.18	1.77	0.220
13	age+g+D$_{(Mus_spr + Cr_rus)}$/ Ad_{res}	.	5	286.72	569.69	7.47	0.01	0.04	0.32*	0.585
14	age+g+D$_{(Cr_rus)}$	g	6	285.31	570.37	8.14	0.00	0.13	1.19	0.307
15	age+g	g	5	287.43	570.4	8.18	0.00			

16	age+g+D$_{(Apo_fla + Myo_gl)}$	.	5	287.43	570.4	8.18	0.00	0.02	0.19	0.674
17	age+g+D$_{(Myo_gl)}$	.	5	287.58	570.55	8.33	0.00	0.01	0.11	0.749
18	age+g+D$_{(Apo_fla)}$	.	5	287.7	570.67	8.45	0.00	0.01	0.05	0.829
19	age+g+t	.	13	271.36	571.33	9.11	0.00			
20	g	.	3	292.48	571.34	9.12	0.00			
21	age+g+t/ Ad$_{res}$	.	14	269.22	571.38	9.16	0.00			
22	age+g+D$_{(Mus_spr + Cr_rus)}$/ Ad$_{res}$	.	6	286.38	571.43	9.21	0.00	0.06	0.48*	0.506
23	age+g+D$_{(Apo_fla + Myo_gl)}$	g	6	287.03	572.08	9.86	0.00	0.02	0.20	0.667
24	age+g+D$_{(Myo_gl)}$	g	6	287.18	572.23	10.01	0.00	0.02	0.12	0.738
25	age+g+D$_{(Apo_fla)}$	g	6	287.33	572.39	10.17	0.00	0.01	0.04	0.846
26	g	g	4	291.97	572.88	10.66	0.00			
27	age+g+t/ Ad$_{res}$	g	15	268.85	573.22	10.995	0.00			
28	age+g+t	g	14	271.09	573.25	11.03	0.00			
29	age	.	3	294.69	573.55	11.33	0.00			
30	age	g	4	293.66	574.57	12.35	0.00			
31	g+t	.	12	278.30	576.10	13.88	0.00			
32	age+t	.	12	278.49	576.29	14.07	0.00			
33	(age+g)*t	.	20	261.51	577.11	14.89	0.00			
34	age+t	g	13	277.62	577.59	15.37	0.00			
35	g+t	g	13	277.93	577.90	15.68	0.00			
36	(age+g)*t	g	21	260.86	578.75	16.53	0.00			
37	t	.	11	289.98	585.63	23.40	0.00			
38	t	g	12	288.48	586.28	24.06	0.00			
39	(age+g)*t	t	26	260.66	590.25	28.02	0.00			
40	(age+g)*t	g*t	31	249.69	591.38	29.16	0.00			
41	(age+g)*t	g+t	27	259.67	591.65	29.43	0.00			

*Here, degrees of freedom are $F_{(1,9)}$
